# Bioelectrocatalyst for O_2_ Reduction Based on a Novel Recombinant Two-Domain Laccase from *Streptomyces ochraceisleroticus* Immobilized on Naphthyl-Modified MWCNTs

**DOI:** 10.3390/ijms26189143

**Published:** 2025-09-19

**Authors:** Liubov Trubitsina, Konstantin Egorov, Azat Abdullatypov, Marina Petrakova, Ivan Trubitsin, Sergey Alferov, Alexey Leontievsky, Olga Ponamoreva

**Affiliations:** 1 G.K. Skryabin Institute of Biochemistry and Physiology of Microorganisms, Federal Research Center “Pushchino Scientific Center for Biological Research of the Russian Academy of Sciences”, 142290 Pushchino, Russia; 2 Department of Biotechnology, Tula State University, 300012 Tula, Russias.v.alferov@gmail.com (S.A.); 3 Institute of Basic Biological Problems, Federal Research Center “Pushchino Scientific Center for Biological Research of the Russian Academy of Sciences”, 142290 Pushchino, Russia; azatik888@yandex.ru; 4 Laboratory of Ecological and Medical Biotechnology, Tula State University, Friedrich Engels Street 157, 300012 Tula, Russia

**Keywords:** two-domain laccase, *Streptomyces ochraceiscleroticus*, heterologous expression, bioelectrocatalysis, direct electron transfer, oxygen reduction reaction, redox potential, modified MWCNT

## Abstract

A novel two-domain small laccasefrom *Streptomyces ochraceiscleroticus* (SoSL) was produced through recombination in *Escherichia coli* and purified by affinity chromatography. The properties (thermal optimum and thermostability, pH optima and pH-stability), kinetic characteristics, substrate specificity and dye decolorization ability were estimated. Laccase SoSL was able to oxidize 2,2′-azino-bis (3-ethylbenzothiazoline-6-sulfonic acid (ABTS)and 2,6-dimethoxyphenol (2,6-DMP) with at a maximal rate at pH 3.5 and 9.0, respectively, and was stable at pH 9.0 (retained 75% activity after incubation at room temperature for 120 h). High enzyme affinity to ABTS is caused by an expanded area occupied by aromatic amino acid residues on its surface. Substrate-directed immobilization of the enzyme was performed using naphthylated multiwalled carbon nanotubes (MWCNTs), and a high oxygen reduction reaction potential (+0.62 V vs. normal hydrogen electrode (NHE)) was observed. The above-mentioned features make this enzyme a promising one for further studies in bioremediation and biological fuel cell technologies.

## 1. Introduction

Laccases (benzenediol–oxygen oxidoreductases, EC 1.10.3.2) are multicopper oxidases with active sites consisting of four copper atoms organized into a mononuclear cluster (T1 copper center with one copper atom) and a trinuclear cluster composed of a T2 metal center (one copper atom) and T3 metal center (two copper atoms). Laccases catalyze the oxidation of a wide range of phenolic and non-phenolic compounds. Their final electron acceptor in such reactions is molecular oxygen, which is reduced to water in a four-electron process.

Laccases are widespread in fungi, plants, and bacteria. Particularly, they are present in numerous soil actinobacteria belonging to the genus *Streptomyces*. From the point of view of domain organization, laccases are divided into two-domain and three-domain enzymes. Small two-domain laccases (SLACs, or 2D-laccases) consist of two cupredoxin domains. The first identified SLAC was isolated from *Streptomyces coelicolor*. All SLACs (2D laccases) are resistant to sodium azide (NaN_3_) and fluoride (NaF), and they are active at alkaline pH values [1]. The mechanisms responsible for the distinguishable behavior of 2D laccases are still unclear. Two-domain laccases are mostly thermostable enzymes capable of oxidizing phenolic substrates in neutral and alkaline media. These laccases are supposed to participate in the process of humification [2,3], in lignin degradation [4,5,6] and in morphogenesis [7].

The application of laccases in various industries leads to expansion of the market of these enzymes. Today, the estimated size of laccase market is more than USD 3 billion (custommarketinsights.com/report/laccase-market/, accessed on 10 September 2025).

Based on numerous studies, a classification of laccases was proposed based on their redox potential. A certain dependence was found: the presence of phenylalanine as an axial ligand of T1 center of laccase indicates high redox potential, leucine and isoleucine are specific for middle-potential enzymes, and methionine confers low redox potential. According to this classification, all the bacterial two-domain laccases were categorized as low-potential enzymes. Indeed, the value of oxidation-reduction potential (ORP) of the characterized two-domain laccases varied from 0.35 to 0.45 V. The low potential of laccases, as was supposed earlier, was caused by the presence of methionine in the axial ligand position in the T1 copper center [8]. However, earlier, we obtained and characterized two two-domain actinobacterial laccases, ScaSL and CjSL, which have methionine as an axial ligand while possessing the middle redox potential [9,10,11]. Site-directed mutagenesis showed that the elevation of redox potential is due to presence of a histidine residue in the 286 position in ScaSL laccase. The presence of threonine (like in laccase CjSL and ScaSL mutant H286T) also characterizes this enzyme as a middle-potential one.

One of the most important features of laccases is their ability for direct electron transfer (DET) in bioelectrochemical systems, as first demonstrated by Russian scientists [12,13]. Laccase has been regarded as an important candidate for the role of a cathodic biocatalyst because the only by-product in the oxygen reduction reaction (ORR) is water, which has almost no harmful effect on the electrode or other components of electrochemical cell or fuel cell assembly [14].

The possibility of DET allows one to determine the redox potentials (E^0′^) of its copper centers, which is crucial for deciphering its catalytic mechanism and directed engineering [15]. It should be noted that for almost five decades of laccase biocathode construction, the vast majority of the studies have used high-potential fungal enzymes [14,16]. Fungal laccases are three-domain proteins active in monomeric form. In contrast, two-domain laccases demonstrate catalytic activity in trimeric forms. The trimeric architecture and relative proximity of type 1 copper atoms to the surface arrange three substrate-binding parts of the monomer. Actually, a shallow substrate-binding pocket with a depth of around 8 Å is formed on the surface of the trimer [17]. During the intramolecular electron transfer, the T1 center of one monomer and T2–T3 cluster of another monomer are involved in the process. A few studies are dedicated to electrochemical processes in SLAC bioelectrocatalysis [18,19,20,21]. The possibility of DET during SLAC bioelectrocatalysis has been shown, and it depended on the type and size of the carbon nanomaterials. The oxygen reduction onset potential was shown to be higher than the redox potential of the T1 copper atom determined by redox titration. Immobilization of SLAC on pyrene and the neocuproine promoter-modified graphite allowed for the appropriate immobilization of the laccase to elevate the degree of DET. Thus, bacterial laccases have certain structural advantages: accessible T1 center, which shortens the path of electron transfer; thermal stability; ability to function in neutral and alkaline media; resistance to halide inhibition. All these advantages might promote their application in oxygen reduction bioelectrocatalysis in biofuel cell application.

In the current work, we present the results of experimental and computational studies of a novel two-domain small laccase from *Streptomyces ochraceiscleroticus* VKM Ac-651. The enzyme was expressed in a heterologous bacterial expression system, and it was shown to be a high-affinity catalyst of ABTS oxidation and a promising bioelectrocatalyst of oxygen reduction when immobilized via substrate-like modification of carbon nanotubes. The results of the experiments are supported by homology modeling and docking computations.

## 2. Results

### 2.1. Expression and Purification of the SoSL Enzyme

**Figure 1:**
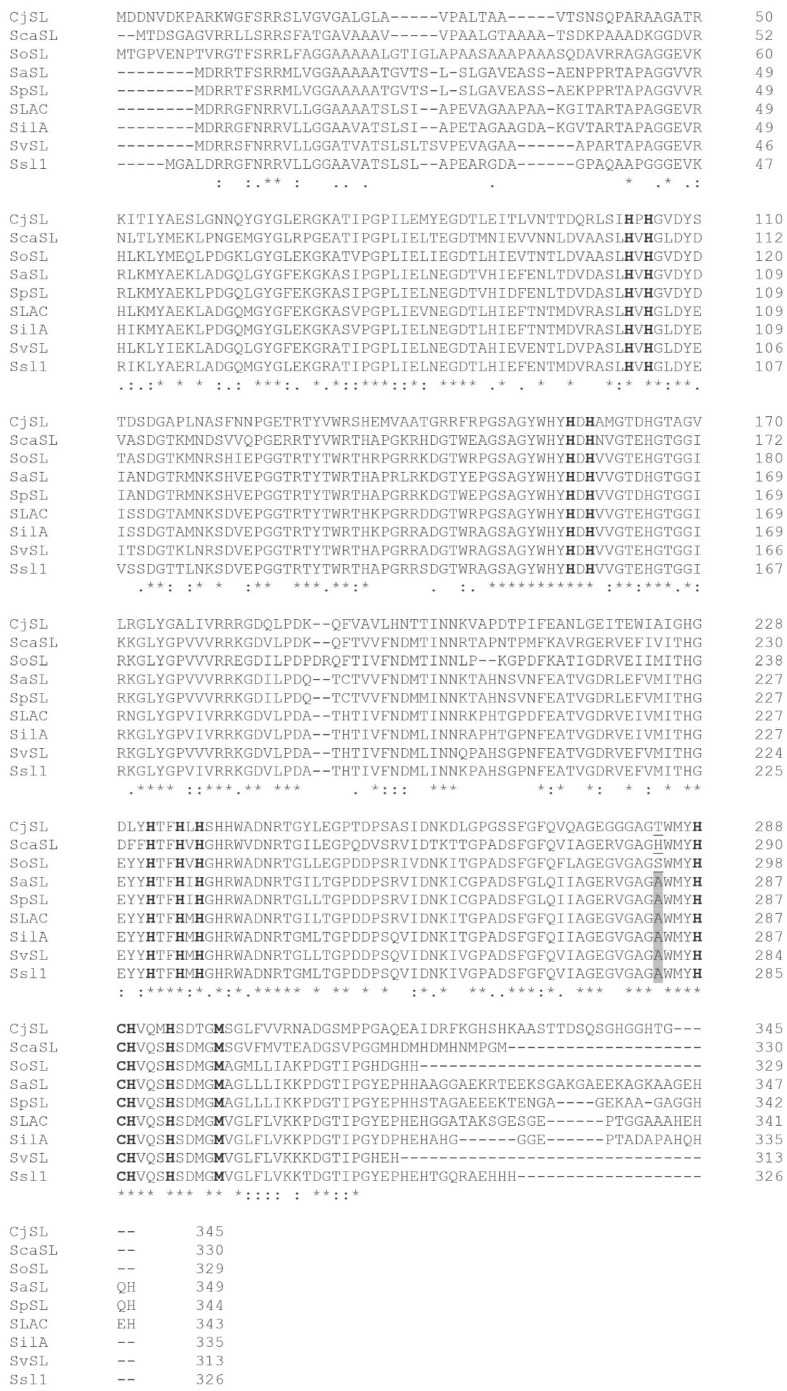
Alignment of the studied two-domain laccases of actinobacteria. Conservative histidine and cysteine residues and axial T1 copper ligand (methionine) are shown in bold type. Gray color highlights the alanine residues specific for low-potential enzymes. *—identical residues; :—strongly conserved substitution; .—weakly conserved substitution (according to BLOSUM62 substitution matrix).

The *Streptomyces ochraceiscleroticus* genome has a gene encoding a two-domain laccase with a twin-arginine translocation (TAT) signal sequence and conservative copper-binding residues designated here as SoSL. The alignment of the amino acid sequence of this laccase with the low- and middle-potential laccases did not allow us to assign it to one of these groups because the position corresponding to alanine A283 specific for low-potential laccases is occupied by serine S294, i.e., neither by threonine nor by histidine, which are attributed to middle-potential laccases (Figure 1).

Since the position corresponding to alanine in low-potential laccases is occupied by serine (neither by threonine nor by histidine specific for middle-potential laccases), it was impossible to classify it by redox potential without experimental study.

The gene encoding the two-domain laccase designated as SoSL was cloned from the *Streptomyces ochraceiscleroticus* VKM Ac-651 in pQE-30 vector. The cloned bacterial laccase gene encoded a protein of 329 amino acids. Analysis of the amino acid sequence of SoSL showed the presence of a signal peptide (1–36, 1–39 or 1–40) for the translocation of laccase along the TAT pathway and two cupredoxin domains with conservative copper-binding amino acids: ten histidines and one cysteine. Three variants of the recombinant laccases were obtained: one of them lacked 36 *N*-terminal amino acids, another one lacked 39 Aa, and the third one lacked 40 *N*-terminal residues. All the three laccases had enzymatic activity, but the enzyme without 40 amino acid signaling moiety had no blue color specific for the laccases. Moreover, the enzymes without 39 and 40 amino acids had lower enzymatic activity compared to the variant without 36 amino acid residues. Due to that, all the further experiments were performed on the enzyme with the deletion of 36 *N*-terminal residues.

**Figure 2:**
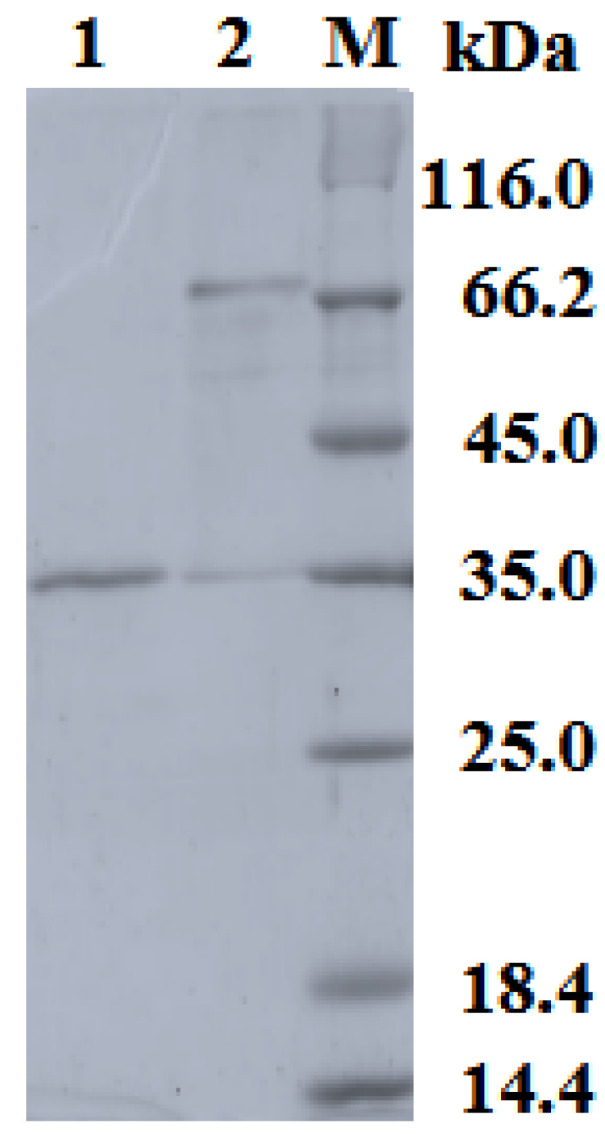
SDS-PAGE of laccase from *S. ochraceiscleroticus* VKM Ac-651. (M)—molecular weight markers; (1)—purified enzyme boiled with β-mercaptoethanol and SDS; (2)—purified enzyme with β-mercaptoethanol and SDS but without boiling.

The yield of the protein from the bacterial culture was around 35 mg/L. The theoretically computed molecular mass of the laccase was 33 kDa. According to the SDS-PAGE electrophoresis (Figure 2), the molecular weight of the monomeric protein was around 35 kDa, which is consistent with the molecular weight of the protein calculated on the basis of the protein sequence.

**Figure 3:**
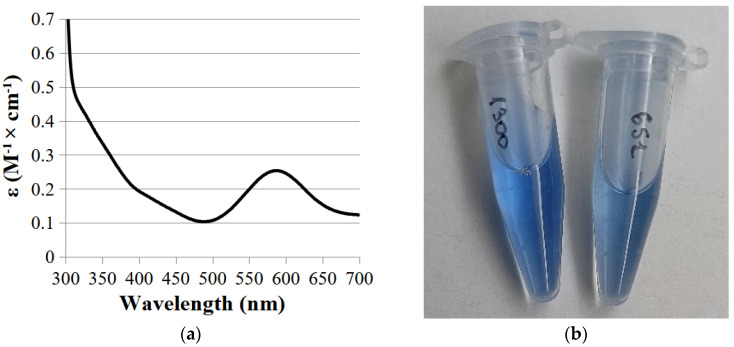
Absorption spectra of SoSL (**a**) and color of the purified laccases ScaSL (Ac-1300, left) and SoSL (Ac-651, right) (**b**).

The UV-visible spectrum of laccase had an absorption peak at 590 nm due to the presence of copper ion in the T1-active center and the shoulder at 330 due to the presence of the T3-active copper center (Figure 3a).

The color of the obtained enzyme differed from the two-domain laccases obtained earlier in our works (Figure 3b).

### 2.2. Biochemical and Computational Characterization of SoSL

**Figure 4:**
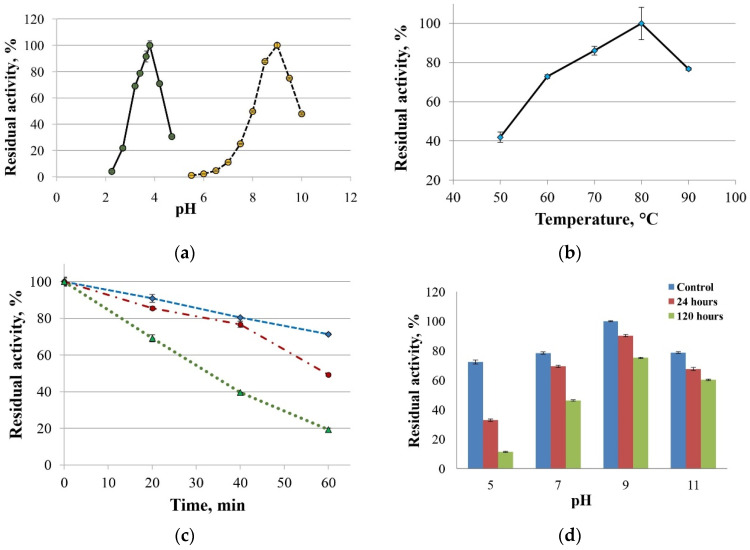
Biochemical properties of SoSL. (**a**)—optimum pH with ABTS and 2,6-dimethoxyphenol; (**b**)—effect of temperature on laccase activity; (**c**)—thermal stability at 70 °C, 80 °C and 90 °C (blue, red and green line, respectively); (**d**)—pH stability of laccase.

The maximal ABTS oxidation rate was observed at pH 3.8, and maximal laccase activity towards 2,6-DMP was seen at pH 9.0 (Figure 4a). The optimum reaction temperature for the SoSL laccase was 70 °C (Figure 4b). The enzyme was more stable at pH 9–11. It retained 75% and 60% of its initial activity after incubation for five days at pH 9 and pH 11, respectively (Figure 4c). The residual laccase activities after one hour of incubation at 70 °C, 80 °C, and 90 °C were 71%, 49% and 19%, respectively (Figure 4d).

Qualitative assessment of the enzyme’s substrate specificity showed that SoSL was able to oxidize typical phenolic substrates: caffeic acid, ferulic acid, gentisic acid, 2,6-dimethoxyphenol, 2-aminophenol, 4-chloro-1-naphthol, catechol, guaiacol, hydroquinone, pyrogallol, methylhydroquinone, and syringaldazine (Table 1).

The kinetic constants of SoSL were determined with ABTS (pH 3.5) and 2,6-DMP (pH 9.0). For ABTS, the *K_m_* was 0.05 mM; *V_max_* = 62 nmol/min; *k_cat_* = 106 s^−1^. For 2,6-DMP, the *K_m_* was 0.76 mM; *V_max_* = 1.02 nmol/min; *k_cat_* = 0.11 s^−1^. Such a high affinity towards ABTS is unusual for bacterial two-domain laccases.

Table 2 lists comparative Michaelis constants for five different actinobacterial two-domain laccases available in the laboratory and the calculated binding constants derived from docking affinity scores. In order to achieve more realistic results, after the initial docking performed on the homology models, the models were then subjected to energy minimization at pH values equal to ABTS oxidation optima in YASARA Structure and then used in docking calculations in Autodock VINA again.

**Figure 5:**
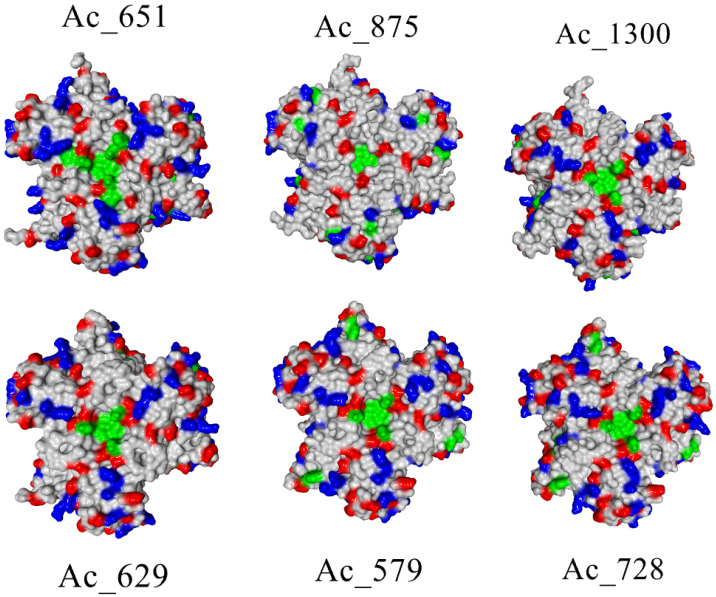
Molecular surfaces of six actinobacterial laccases (view from the side of T1 center). Negatively charged amino acid residues (Asp, Glu) are shown in red, positively charged residues (Lys, Arg) are shown in blue, aromatic residues (Phe, Tyr, Trp) are shown in green, all other residues are shown in grey.

It can be clearly seen that the Michaelis constant does not correlate with docking affinities independent of pH correction and energy minimization made for the protein molecule. However, docking calculations regarded the laccase molecules as rigid ones, without any flexibility of possible substrate-binding residues taken into consideration. So, the reason for the high affinity towards ABTS should be in the fine structure of substrate-binding residues close to the T1 center. To assess their distribution in order to judge the possible roles of certain groups in the interactions, colored surfaces of laccases were built, where positively charged, negatively charged and aromatic residues were highlighted in different colors (Figure 5).

**Figure 6:**
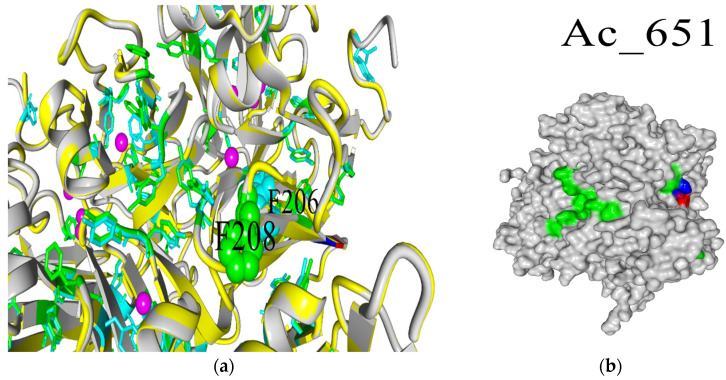
(**a**) Aromatic surfaces of SoSL (shown in green) and unique DR201-202 moiety (shown in red and blue). (**b**) Positions of F208 (SoSL, green spheres) and F206 (SaSL, cyan spheres) in the structural alignment of two enzymes. Magenta spheres represent copper atoms; yellow trace is the main chain of SaSL; gray trace is the main chain of SoSL; red and blue denote DR moiety of SoSL.

The surface view of the T1 center region of laccases shows that SoSL (Ac651) possesses the largest area occupied by aromatic residues near the active site. This could be the reason for the favorable interaction with ABTS; however, there are no clearly definable determinants of such a feature in the amino acid sequence because this surface is formed by tyrosine residues 240–241 and phenylalanine 208 (numbering according to the alignment in Figure 1), which are quite conservative among the laccase set chosen for this study. So, the most probable reason for such an area of aromatic surface could be the sterical effect of DR moiety (DR201-202) unique to the SoSL, which could have a long-acting conformational effect because it is located on the side of laccase (Figure 6a). The interaction between DR moiety and aromatic amino acids is likely due to the increased length of the polypeptide chain causing some distortions. For example, it can be clearly seen from the position of F208 when compared with corresponding F206 from Ac-728 (SaSL) (Figure 6b).

So, the hypothesis for the reason behind the affinity of SoSL towards ABTS is its large area of aromatic residues in the T1 region. Meanwhile, this is caused not by an elevated number of aromatic residues but rather by structural change caused by the insertion of DR moiety in the primary sequence.

The main advantage of two-domain laccases over three-domain enzymes is their high tolerance to inhibition by fluoride ions. For example, the laccase from *Catenuloplanes japonicus* was intolerant to fluoride inhibition at alkaline pH values [11]. The activity of SoSL was also assessed in the presence of fluoride ions (Table 3).

It is known that fluoride ions inhibit mainly the T2/T3 three-core copper cluster. In an alkaline environment, where phenols are best oxidized by 2D laccases, fluoride is in the ionic form of F^−^ and cannot effectively reach the cluster through a hydrophobic tunnel, so there is no inhibition. In an acidic environment, fluorides exist mainly in the form of hydrofluoric acid HF, which is a much more effective inhibitor due to its ability to penetrate to the active site. This accounts for the inhibitory effect of fluoride on ABTS oxidation.

### 2.3. Bioelectrocatalysis

**Figure 7:**
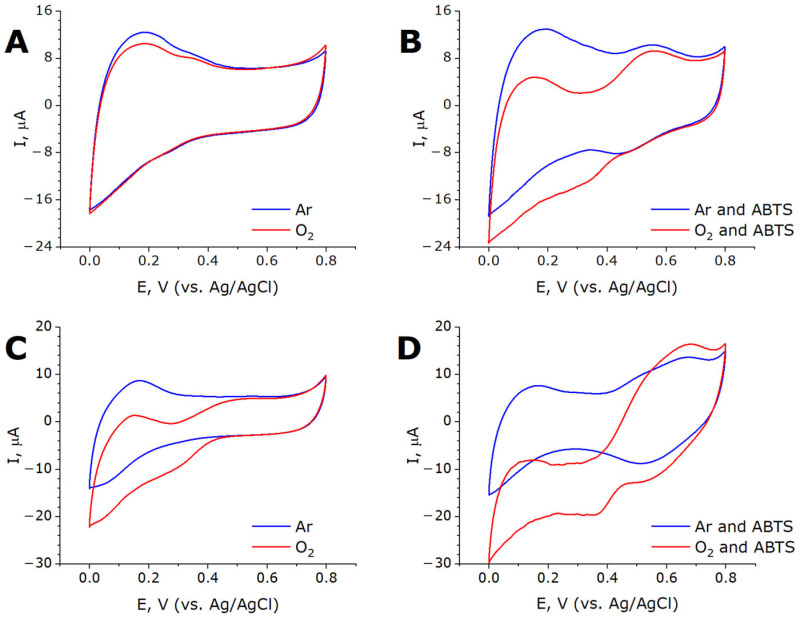
Cyclic voltammograms of MWCNT-SoSL electrodes in air (red lines, O_2_) and argon (blue lines) atmosphere. Upper line (**A**,**B**), MWCNTox-SoSL; lower line (**C**,**D**), MWCNTnaph-SoSL. Left column (**A**,**C**), CV in absence of ABTS; right column (**B**,**D**), CV in presence of ABTS.

Electrodes modified with MWCNTox-Lac and MWCNTnaph-Lac were tested by cyclic voltammetry to reveal the capability of laccase for direct electron transfer and molecular oxygen reduction. In the systems based on MWCNTox-Lac, the cyclic voltammograms in the presence and absence of oxygen did not differ from each other, and the direct electron transfer from the electrode to laccase was absent (Figure 7A). Upon the addition of ABTS into the electrochemical cell in aerobic conditions, a cathodic wave appears, which could be related to the biochemical activity of the adsorbed laccase (Figure 7B). In contrast to MWCNTox-Lac, MWCNTnaph-Lac electrode cyclic voltammetry showed the onset of an oxygen reduction reaction at +0.44 V vs. Ag/AgCl (Figure 7C). Thus, the onset of oxygen reduction begins at +0.64 V vs. NHE, which is significantly higher than could be expected from usual low-potential two-domain laccases. Control experiments with MWCNTnaph electrodes without laccase displayed only background current. This means that the enzyme is oriented on the electrode surface via the interaction of the T1 center with substrate-like naphthyl substitutors, providing the direct electron transfer (DET). In the presence of ABTS, anodic and cathodic peaks at +0.68 V and +0.51 V (vs. Ag/AgCl), respectively, are observed in both the presence and absence of oxygen (Figure 7D).

In the presence of oxygen, the cathodic current increases significantly due to the increased content of ABTS^+^ via the biochemical oxidation of ABTS by the substrate-exposed T1 centers of the enzyme. In this case, ABTS as a redox mediator provided mediated electron transfer (MET). The difference in DET and MET currents at 0 V (vs. Ag/AgCl) reflected the share of properly oriented laccase molecules capable of DET (Equation (1)):

ω = (ΔI_DET_/ΔI_MET_) × 100% (1)

where ω is the share of properly oriented laccase molecules, %;

ΔI_DET_ is the difference in direct electron transfer current in air and in an argon atmosphere, μA;

ΔI_MET_ is the difference in mediated electron transfer current in air and in an argon atmosphere, μA.

The share of properly oriented enzyme molecules was equal to 56%.

**Figure A1:**
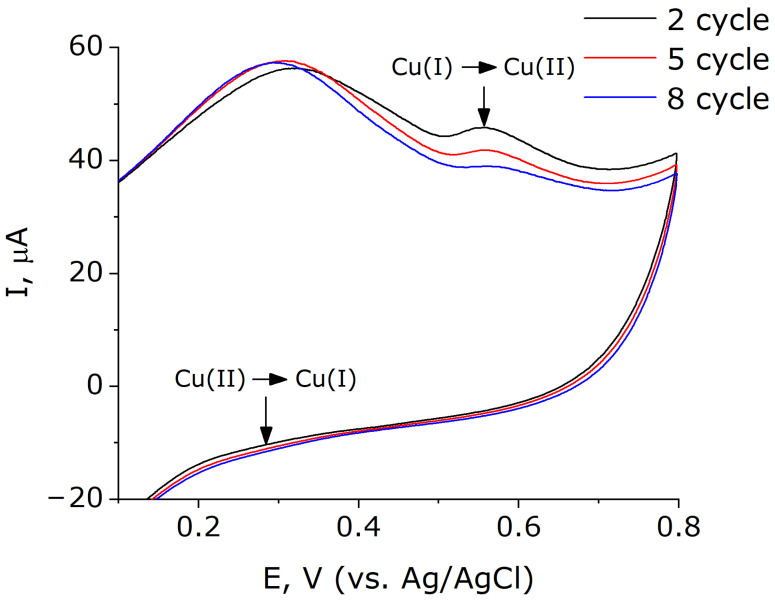
Cyclic voltammogram of SoSL-MWCNTnapth in anaerobic conditions showing redox transformations of copper.

It should be noted that the voltammograms registered with MWCNTnaph-Lac electrodes revealed weak anodic peaks at initial cycles (+0.56 V vs. Ag/AgCl) and cathodic peaks at +0.26 V (vs. Ag/AgCl) (Appendix A Figure A1). The appearance and amplitude of these peaks is affected by the number of voltammetry cycles, potential sweep rate, and presence of oxygen in the electrochemical cell.

**Figure A2:**
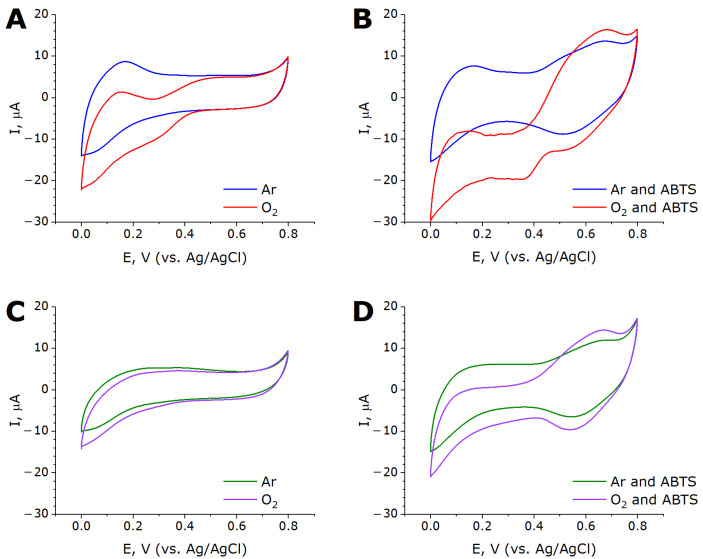
Cyclic voltammograms of MWCNTnapth-SoSL electrodes. (**A**,**B**)—intact electrodes; (**C**,**D**)—electrodes after heating at 90 °C. (**A**,**C**)—electrodes in absence of ABTS; (**B**,**D**)—electrodes in presence of ABTS.

To confirm the leading role of laccase in bioelectrocatalysis, the working electrodes were heated at 90 °C for an hour to inactivate the enzyme (Appendix A Figure A2).

After heating the electrodes, the oxygen reduction current at 0 V decreased two-fold, corresponding to 50% residual activity of the immobilized laccase SoSL. The decreased reduction current after partial inactivation of the enzyme confirms the participation of SoSL in both direct and mediated electron transfer. Moreover, the immobilization of laccase on MWCNTnaph elevates its thermal stability compared to the enzyme in solution (19% residual activity after the same heating time in solution).

During the electrochemical measurements, the electrodes remained stable at room temperature throughout the experiment. The number of cycles with no significant reduction in the peak magnitude was at least 8. The electrodes were also stable when stored at +4 °C for at least one week, after which they were no longer used. The enzyme in the buffer solution remained active for at least three months.

**Figure 8:**
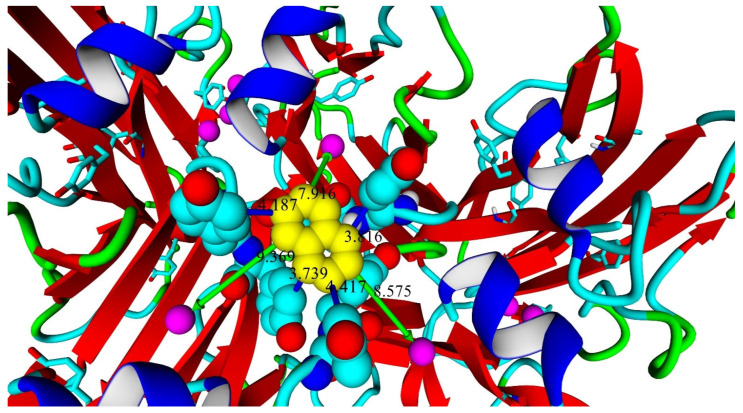
Position of naphthalene (yellow) in the complex with Ac651 shown by molecular docking. Green arrows show distances from naphthalene to T1 copper atoms (9.369, 7.916 and 8.575 Å); blue sticks show contacts between naphthalene and aromatic carbon atoms of tyrosine residues (4.187, 3.816, 4.417 and 3.739 Å). Standard color scheme is used for atom designation: cyan, carbon; red, oxygen; blue, nitrogen; magenta, copper. Secondary structure elements: blue, alpha-helices; red, beta-sheets; green, turns; cyan, coils.

Molecular docking of naphthalene to the T1 center of the enzyme showed that it could be located in such a manner that electron transfer could be possible: it interacts mainly with tyrosine residues close to T1 copper centers (Figure 8).

Therefore, the results of naphthalene docking allow us to suppose that the naphthyl groups of MWCNTnaph electrodes are interacting with aromatic residues in close proximity to the T1 center of laccase, enabling direct electron transfer. The effective substrate-directed immobilization of SoSL is correlated with the high affinity of the enzyme toward ABTS and could hypothetically be due to the influence of DR moiety (DR201-202), which is unique to the SoSL.

The weak peaks observed on the initial cyclic voltammograms of MWCNTnath-Lac bioelectrodes (+0.56 V vs. Ag/AgCl, oxidation; +0.26 V vs. Ag/AgCl, reduction) in the absence of oxygen (see Appendix A Figure A1) can be attributed to redox processes in the T1 copper center of the immobilized laccase SoSL. Similar CV shapes were observed in the studies on intramolecular electron transfer in fungal laccases [22,23,24,25,26] and “yellow” bacterial laccase [20], using electrochemical methods. The half-wave potential of these peaks is equal to +0.61 V vs. NHE, and it is attributed to the redox potential of T1 center bound to naphthyl substitutors of MWCNTnaph.

## 3. Discussion

The characterized laccase SoSL from Streptomyces ochraceiscleroticus possesses two practically interesting features. One of them is its high affinity towards ABTS. ABTS is a widely used redox mediator that enhances the ability of laccases to oxidize various pollutants, such as dyes [27,28,29], polyaromatic pollutants [30,31], antibiotics [32,33,34] and biogenic toxins [35,36,37]. The mechanism of ABTS action is the formation of a highly active radical (ABTS*) capable of oxidizing various substrates and promoting further radical processes.

ABTS is quite an expensive substance to use in large-scale applications. Hence, when designing a novel bioremediation system based on laccase–ABTS interaction, the possibility of reducing the amounts of the mediator in use should be kept in mind, so that the system should be economically feasible. To reduce ABTS consumption per pollutant unit, various strategies could be applied. One of the strategies could lie in the application of laccases with a low Michaelis constant, so that the working concentration of ABTS could be low. Indeed, SoSL laccase definitely has a low Michaelis constant compared to other actinobacterial laccases. It is comparable to the widely used commercial *Trametes versicolor* laccase with a *K_m_* of 56 μM [38], and its alkaline pH stability and thermostability might way to practical applications.

The application of an ABTS–laccase system in organic synthesis may be more promising because of the lower limitation of ABTS costs, considering the high value of the derived products. One such application could be the synthesis of conductive polymers such as polypyrrole; for example, the work by Song and Palmore on the enzymatic polymerization of pyrrole resulted in the formation of polypyrrole film containing trace amounts of ABTS functioning as a dopant [39]. Since a dopant is a substance that should be present in the resulting polymer in trace amounts, experiments with low concentrations of ABTS are of interest, providing the requirement for use of laccases with low *K_m_*.

Another distinguishable feature of SoSL, its high value of oxygen reduction onset potential (at least for two-domain laccases) demonstrated in bioelectrochemical measurements, also makes it a promising enzyme for application in biofuel cells (BFCs). The biological fuel cells based on multicopper oxidases are characterized by several parameters: (1) operational voltage; (2) current density. The higher these parameters are, the more power is produced by the fuel cell, and the operational voltage particularly depends on the onset potential of the biocathode; however, the highest current density values were shown using a low-potential laccase from *Rhus verniciflua* in a methanol fuel cell [40]. In this regard, the high *k_cat_* value of SoSL for ABTS oxidation might render a correspondingly high electrocatalytic activity and allow it to produce high-current-density electrodes in future studies.

The onset potential of a laccase cathode, in turn, depends on the redox potential of the T1 center of the laccase. We suggest that the anodic and cathodic peak currents registered on the CVs of the MWCNTnaph-Lac electrode in argon at +0.56 and +0.26 V, respectively (see Appendix A Figure A1), correspond to the redox processes in the T1 copper center. Similar CV peaks were observed in studies of electron transfer in plant [41], fungal [22,23,24,25,26] laccases, and “yellow” bacterial laccase [20] by electrochemical analysis methods.

**Figure A3:**
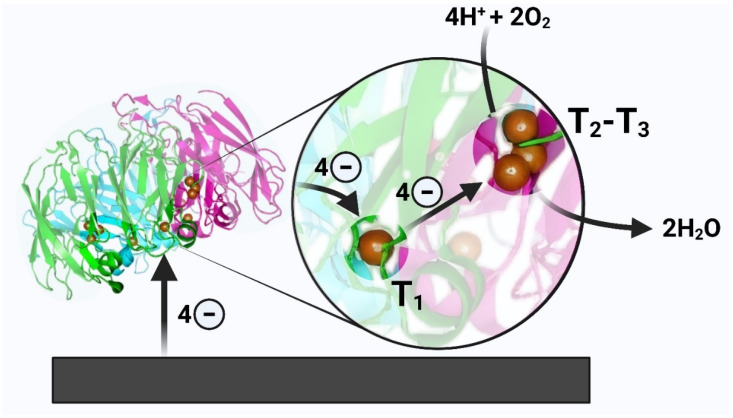
A scheme of electron transfer in trimeric two-domain laccase immobilized on carbon electrode. Three subunits are highlighted in green, cyan and magenta.

An interesting feature of two-domain laccases is the route of electron transfer (see Appendix A Figure A3). The electrons from the T1 center of one protein monomer can be transferred to the T2/T3 copper atoms of another monomer (the T2/T3 coppers are located at the interfaces between the monomers, so the histidine amino acid residues of both subunits participate in the anchoring of these metal ions).

Johnson et al. made a detailed cyclic voltammetry study of electron transfer from *Rhus vernicifera* laccase covalently attached to golden electrodes. They showed that first scans at high sweep rates (above 50 mV/s) demonstrated a large anodic peak, which was four times greater than the corresponding cathodic peak. The authors attributed it to different rates of oxidation and reduction of the T1 center [41].

Another work on the cyclic voltammetry of laccase from *Polyporus versicolor* adsorbed on pyrolytic graphite demonstrated weak oxidation peaks visualized more like shoulders [42], just like the case of the cathodic peak of SoSL laccase in our work (see Appendix A Figure A1). The redox potential of the SoSL T1 center is significantly higher than that of other bacterial 2D laccases. For example, the SLAC enzyme from *Streptomyces coelicolor* showed a redox potential of +0.42 V vs. NHE when adsorbed on a carbon electrode surface [18]. However, *S. coelicolor* SLAC is a low-potential enzyme with a clearly distinguishable determinant of the low redox potential (see the highlighted alanine residue in Figure 1), while SoSL behaves more like a middle-potential enzyme in the bioelectrocatalytic experiments.

The calculated maximal current density of DET of the MWCNTnaph-SoSL electrode calculated at +0.15 V vs. Ag/AgCl, 50 mV/s sweep rate and pH 3.9 comprises 0.59 mA/cm^2^ (subtracting the background current 0.17 mA/cm^2^), which is comparable to data obtained for *Streptomyces coelicolor* laccase [19] at the same sweep rates and potential values. These values are still too low for practical applications; however, the current density depends on many factors (electrode type, its actual area, enzyme load, enzyme activity, etc.) and the optimization of these factors could lead to significantly higher current values.

In spite of the modest current density, the SoSL bioelectrode demonstrated a high oxygen reduction onset potential and high thermal stability, making it a good candidate for further studies and biocathode development.

## 4. Materials and Methods

### 4.1. Microorganism Cultivation, Gene Cloning, Protein Purification

Strain *S. ochraceiscleroticus* VKM Ac-651 was obtained from the all-Russian collection of microorganisms (https://vkm.ru/rus/, accessed on 1 March 2024). Cultivation of bacteria, two-domain laccase gene cloning, expression and protein purification were identical to those previously described [9], except for cultivation temperature after induction (16 °C) and inducer concentration (0.25 mM). Primers for PCR: 651F 5′-ATGACCGGTCCAGTCGAAAACC-3′ and 651R 5′-TCAGTGATGCCCGTCGTG-3′ were designed based on the predicted *S. ochraceiscleroticus* VKM Ac-651 multicopper oxidase nucleotide sequence (NCBI Reference Sequence of protein: WP_031060324.1). The laccase gene was amplified from genomic DNA, and the gene sequence was confirmed by sequencing. To clone the laccase gene into the pQE-30 expression plasmid, it was amplified using the following primers:

651Fe40nS 5′-AGTGGATCCGCACCGGCCGCCGCGTCC-3′;

651Fe36nS 5′-AGTGGATCCGCCTCGGCCGCCGCACCG-3′;

651Fe39nS 5′-AGTGGATCCGCCGCACCGGCCGCCGCG-3′;

651Re 5′-AGTAAGCTTTCAGTGATGCCCGTCGTG-3′.

The use of these primers made it possible to obtain recombinant laccases lacking 40, 39 and 36 *N*-terminal amino acids, respectively. Expression of the proteins was performed in the strain *Escherichia coli* M15 [pREP4]. After the chromatography stage (HisTrapTMFF column (GE Healthcare, Chicago, IL, USA) with Ni-sepharose), the blue fractions containing purified laccase were pooled and dialyzed against 20 mM Tris-HCl buffer (pH 9.0) with 0.1 M NaCl, and then against 20 mM Tris-HCl buffer pH 9.0 without NaCl. The enzyme was stored at 8 °C. Protein concentration was determined using a molar extinction coefficient calculated from an amino acid sequence using the Vector NTI Program (Life Technologies, Carlsbad, CA, USA); SoSL ε_280_ = 41.370 M^–1^ × cm^–1^. Two-domain laccases, ScaSL and SpSL, for a comparative substrate specificity test were obtained as described earlier [3,9].

### 4.2. Characterization of SoSL

Molecular weight of the enzymes was determined with sodium dodecyl sulfate-polyacrylamide gel electrophoresis (SDS-PAGE), according to Laemmli [43]. Presence of conserved domains and TAT-signal sequence was identified in InterProScan (https://www.ebi.ac.uk/interpro/, accessed on 1 March 2024) [44] and SignalP (https://services.healthtech.dtu.dk/services/SignalP-4.1/, accessed on 1 March 2024; https://services.healthtech.dtu.dk/services/SignalP-6.0/, accessed on 1 March 2024) [45,46] services, respectively. Laccase activity was routinely determined at room temperature by the rate of oxidation of 1 mM 2,6-dimethoxyphenol (2,6-DMP) oxidation (ε_469_ = 49.600 M^–1^ ×·cm^–1^) [47] and 0.2 mM 2,2-azino-bis-(3-ethylbenzthiazoline-6-sulfonate) (ABTS) (ε_420_ = 36.000 M^–1^·× cm^–1^) [48] in a 50 mM Britton–Robinson buffer solution [49]. The steady-state kinetic constants were obtained for the substrates 2,6-DMP and ABTS at pH 3.5 and 9.0, respectively, at 30 °C. Calculation of the apparent kinetic constants was performed identically to those previously described [10]. The UV-visible absorption spectrum of laccase was recorded in the wavelength range from 700 to 300 nm on a Shimadzu UV-1800 spectrophotometer (Kyoto, Japan). pH optimum of enzyme activity was determined using 0.2 mM ABTS and 1 mM 2,6-DMP as substrates in 50 mM Britton–Robinson buffer (pH range of 2.5 to 5.5 for ABTS and from 5.5 to 10 for 2.6-DMP). pH stability was estimated at pH values of 5, 7, 9, 11 by incubation of the enzyme at room temperature for 5 days in 100 mM Britton–Robinson buffer and the determination of residual activity in 50 mM Britton–Robinson buffer (pH 3.6) with 0.2 mM ABTS as a substrate. Thermal stability of SoSL was determined by measuring residual activity of the enzyme incubated for 1 h at 70 °C, 80 °C, and 90 °C. Residual activity assay was performed in the same buffer. Optimal temperature was determined using a temperature range from 35 °C to 85 °C in the same buffer with 1 mM 2,6-DMP as a substrate at pH 9.0. Substrate specificity of laccase was examined against 25 different potential substrates by detecting the changes in their absorption spectra after 24 h incubation. The substrate specificity was assayed at pH 9.0 for all compounds in 50 mM Britton–Robinson buffer. The following compounds were analyzed: caffeic acid, coumaric acid, ferulic acid, gallic acid, gentisic acid, syringic acid, tannic acid, vanillic acid, 2-thiobarbituric acid, 3,4-dihydroxybenzoic acid, 4-hydroxybenzoic acid, 2,6-dimethoxyphenol, 3,5-dimethoxyphenol, 3,4,5-trimethoxyphenol, 2-aminophenol, 4-chloro-1-naphthol, catechol, guaiacol, hydroquinone, pyrogallol, methylhydroquinone, syringaldehyde, syringaldazine, vanillin and tyrosine.

### 4.3. Homology Modeling

Homology modeling was performed as described earlier [10]. The models were built in the MODELLER 9.19 program package [50]. SLAC laccase (PDB ID: 3CG8) was used as a template for homology modeling [17].

### 4.4. Molecular Docking

Models of Ac-651 laccase were prepared for docking by the addition of polar hydrogen atoms and Kollman charges in AutodockTools, a part of the MGLTools program package [51]. Ligands (naphthalene and ABTS) were downloaded from the PubChem database [52] and prepared in MGLTools by torsion assignment and merging the non-polar hydrogen atoms. Actual docking computations were performed in AutoDock VINA [53]. A 40-Å docking box was used, and the center of the box was located on the center of mass of T1 copper atoms of the trimeric enzyme.

### 4.5. Graphical Protein–Ligand Complex Representation

YASARA Structure was used for graphical representation of the data after docking calculations [54,55].

### 4.6. Multiwall Carbon Nanotubes Processing and Laccase Modification

Multiwall carbon nanotubes (MWCNT) “Taunit-M” (Nanotechcenter LLC, Tambov, Russia) were produced by CVD synthesis from a propane–butane mixture on a Co/Mo catalyst. According to the manufacturer’s data, their external diameter is 10–30 nm, internal diameter is 5–15 nm, length 2 μm, and specific surface exceeds 270 m^2^/g (determined by BET method [56]).

MWCNTox (5 h of oxidation) was obtained by treating MWCNT “Taunit-M” with chemically pure boiling nitric acid [57].

Functionalization of MWCNTs by naphthyl groups (MWCNTnaph) was performed by their treatment with arene diazonium salts in molten urea [10].

The dispersion of nanotubes was prepared by the following technique: 2.5 mg MWCNTs (MWCNTox or MWCNTnaph) were dispersed in 100 μL of 20 mM Tris-Hcl buffer solution (pH 9.0) in an ultrasonic bath JP-009 (Guangzhou Hanker Electronics Technology Co., Ltd., Guangzhou, China) for 10 min; then, 900 μL of SoSL laccase solution (3.5 mg/mL; 16 units/mg) were added up to 50 units in 1 mL of the dispersion. The mixture was stirred on a VORTEX 2 shaker (IKA, Staufen im Breisgau, Germany) for 2 min at maximal speed and then dispersed in the ultrasonic bath and periodically shaken by VORTEX 2 for 15 min. The suspensions of nanotubes with adsorbed laccase (MWCNTox-Lac, MWCNTnaph-Lac) were stored at +4 °C.

### 4.7. Preparation of Bioelectrodes

The glass–carbon electrode from Zhuhai Minngchuan Technology (Zhuhai, China) (diameter 3.0 mm) was prepared by sanding it with aluminum oxide powder and filter paper, treating it with acetone in a JP-009 ultrasonic bath (Guangzhou Hanker Electronics Technology Co., Ltd., Guangzhou, China) for 15 min, and washing with water and 96% ethanol. Then, 5 μL of laccase-modified MWCNT dispersion was added and dried for 4 h at room temperature. The procedure was repeated three times. After the adsorption of the dispersion, the obtained working electrodes were washed with 20 mM Tris-HCl buffer solution (pH 9.0) and used for electrochemical measurements. Analogously prepared electrodes with MWCNTs without laccase were used as control working electrodes.

To inactivate the adsorbed laccase, the electrodes were kept at 90 °C for 1 h. The degree of enzyme inactivation was assessed by the change in current at 0 V against the Ag/AgCl electrode before and after heat treatment of the electrodes.

### 4.8. Electrochemical Measurements

Voltammetry measurements were carried out using the CS Studio electrochemical station (Corr Test Instruments, Wuhan, China) in a three-electrode electrochemical cell. A modified glass–carbon electrode served as the working electrode, while a filled silver chloride electrode served as the reference electrode. A platinum electrode with dimensions of 1 × 1 × 0.1 cm was used as a counter electrode. The measurements were performed in 20 mM Britton–Robinson buffer (pH 3.9, volume 15 mL).

Cyclic voltammetry was carried out both in an argon atmosphere and in air, with and without 0.1 mM ABTS. The solution was considered saturated with oxygen after bubbling for 15 min with air. Measurements in argon were performed after bubbling with argon for 30 min. To achieve more stable cyclic voltammograms, 8 preliminary cycles were performed in −0.8–+0.8 V range with 50 mV/s sweep rate. Further cycles were performed in 0–+0.8 V range at 10 mV/s sweep rate.

**Figure 9:**
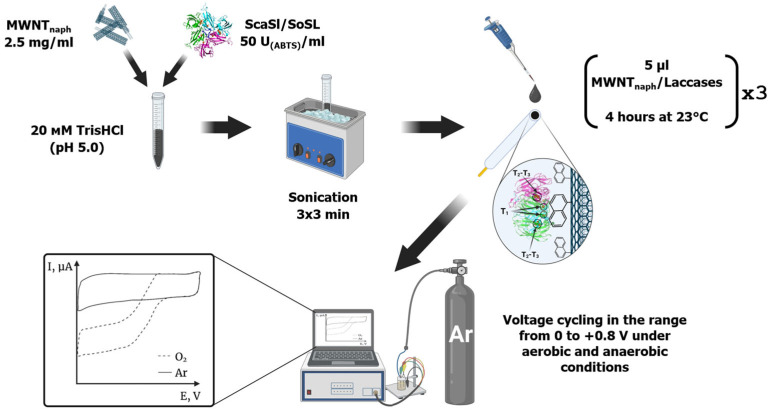
Schematic representation of electrode preparation and electrochemical experiments.

The overall scheme of electrode preparation and analysis is presented in Figure 9.

### 4.9. Statistical Analysis

Mean values and standard deviations were calculated for at least three replicates and the significance criterion was set to *p* < 0.05 (CI = 95%).

## 5. Conclusions

The laccase from *Streptomyces ochraceiscleroticus* was produced in a heterologous *E. coli* system. It displayed high affinity to ABTS, which could be caused by the expanded area occupied by aromatic amino acid residue on its surface. Moreover, it behaved like a middle-potential enzyme (or even high-potential, compared to other bacterial two-domain laccases) in the electrochemical studies. Cyclic voltammetry showed that this enzyme can successfully be immobilized on naphthylated multiwall carbon nanotubes, and molecular docking confirmed the possibility of naphthalene binding in close proximity to T1 copper atoms. The high affinity of SoSL toward ABTS and the effective substrate-directed orientation of the enzyme on naphthylated MWCNTs may be due to the influence of DR moiety (DR201-202) unique to the SoSL, which could have a long-acting conformational effect because it is located on the side of laccase.

The affinity of SoSL towards ABTS can be applied in the future design of other variants of chemically substituted MWCNTs. For example, covalently linked ABTS (or 2-aminobenzothiazole as a “half” of ABTS) are planned to be studied in future experiments with SoSL and carbon nanotubes.

Taken together, the aforementioned features and additionally high thermal stability (higher in immobilized form than in solution) make this enzyme a promising enzyme for further studies in bioremediation and biological fuel cell technologies.

## Figures and Tables

**Table 1:** Oxidation of phenolic compounds by SoSL (Ac-651) and other bacterial laccases.

Substrates	SoSL	SpSL	ScaSL	CjSL
Caffeic acid	+	−	+	+
Coumaric acid	−	−	−	+
Ferulic acid	+	−	+	+
Gallic acid	−	−	+	+
Gentisic acid	+	−	+	+
Syringic acid	−	−	+	+
Tannic acid	−	−	+	+
Vanillic acid	−	−	−	+
2-thiobarbituric acid	−	−	−	−
3,4-dihydroxybenzoic acid	−	−	−	+
4-hydroxybenzoic acid	−	−	−	−
2,6-dimethoxyphenol	+	+	+	+
3,5-dimethoxyphenol	−	−	−	−
3,4,5-trimethoxyphenol	−	−	+	+
2-aminophenol	+	+	+	+
4-chloro-1-naphthol	+	+	+	+
Catechol	+	+	+	+
Guaiacol	+	−	+	+
Hydroquinone	+	+	+	+
Pyrogallol	+	+	+	+
Methylhydroquinone	+	+	+	+
Syringaldehyde	−	−	+	+
Syringaldazine	+	−	+	+
Vanillin	−	−	−	−
Tyrosine	−	−	−	−

**Table 2:** Experimentally determined Michaelis constants and docking-derived binding constants calculated from Autodock VINA affinity scores for the actinobacterial laccases.

Laccase	*K_m_* ABTS, μM	pH Optimum for ABTS	Docking-Derived Binding Constant, μM	Docking-Derived Binding Constant (After Energy Minimization and pH Adjustment of the Receptor), μM
SoSL (Ac-651)	50	3.5	24	5
CjSL (Ac-875)	390	3.6	65	0.06
ScSL (Ac-1300)	100	4.7	9	3
SvSL (Ac-629)	300	4	3	2
SpSL (Ac-579)	370	3.5	2	2
SaSL (Ac-728)	170	3	4	2

**Table 3:** Inhibition of SoSL laccase in presence of sodium fluoride (NaF). The activity is represented as mean ± standard deviation, with control values for each pH taken for 100%.

pH	Control	1 mM	10 mM	100 mM
3.7	100 ± 0.6	84.1 ± 1.2	67.8 ± 0.6	49.4 ± 3 *
8.7	100 ± 0.8	100 ± 2.3	99 ± 1.3	100 ± 0.3

* In this case, the actual concentration of fluoride ion was 80 mM due to dilution of the reaction medium by acid needed to compensate the alkalization caused by hydrogen fluoride.

## Data Availability

Coordinate file of laccase model generated in the study is available at https://doi.org/10.5281/zenodo.16944903.

## Abbreviations

The following abbreviations are used in this manuscript:

2,6-DMP	2,6-dimethoxyphenol
ABTS	2,2′-azino-bis (3-ethylbenzothiazoline-6-sulfonic acid)
DET	Direct electron transfer
MET	Mediated electron transfer
MWCNT	Multiwalled carbon nanotubes
NHE	Normal hydrogen electrode
PAGE	Polyacrylamide gel electrophoresis
SDS	Sodium dodecylsulfate
SLAC	Small laccase
SoSL	*Streptomyces ochraceiscleroticus* small laccase
TAT	Twin-arginine translocation pathway
